# A cyclin-dependent kinase, CDK11/p58, represses cap-dependent translation during mitosis

**DOI:** 10.1007/s00018-019-03436-3

**Published:** 2020-02-06

**Authors:** Sihyeon An, Oh Sung Kwon, Jinbae Yu, Sung Key Jang

**Affiliations:** grid.49100.3c 0000 0001 0742 4007 PBC, Department of Life Sciences, Pohang University of Science and Technology (POSTECH), Cheongam-ro 77, Nam-gu, Pohang-si, Gyeongsangbuk-do 37673 Republic of Korea

**Keywords:** CDK11/p58, Translation initiation, eIF3F, Translational repression in M phase

## Abstract

During mitosis, translation of most mRNAs is strongly repressed; none of the several explanatory hypotheses suggested can fully explain the molecular basis of this phenomenon. Here we report that cyclin-dependent CDK11/p58—a serine/threonine kinase abundantly expressed during M phase—represses overall translation by phosphorylating a subunit (eIF3F) of the translation factor eIF3 complex that is essential for translation initiation of most mRNAs. Ectopic expression of CDK11/p58 strongly repressed cap-dependent translation, and knockdown of CDK11/p58 nullified the translational repression during M phase. We identified the phosphorylation sites in eIF3F responsible for M phase-specific translational repression by CDK11/p58. Alanine substitutions of CDK11/p58 target sites in eIF3F nullified its effects on cell cycle-dependent translational regulation. The mechanism of translational regulation by the M phase-specific kinase, CDK11/p58, has deep evolutionary roots considering the conservation of CDK11 and its target sites on eIF3F from *C. elegans* to humans.

**Electronic supplementary material:**

The online version of this article (10.1007/s00018-019-03436-3) contains supplementary material, which is available to authorized users.

## Introduction

Translation is the last step of gene expression, and regulation of gene expression at the translational level plays important roles in various biological processes owing to its rapid and reversible nature and the necessity of controlling gene expression spatially. Translation is a complex process that requires many protein and RNA participants [1, 2]. Translation initiation requires many translation factors and is the major regulation point.

Global regulation of gene expression often occurs at the translational level for general modulation of cellular physiology, because approximately 50% of cellular energy is consumed during translation and its related reactions [3, 4]. The best known example is stress-dependent repression of translation. Various stresses, such as amino acid starvation, ER overloading, oxidative stress, and viral infection, activate corresponding kinases that phosphorylate the alpha subunit of the translation initiation factor, eIF2, which loads the initiator tRNA (Met-tRNA_i_^Met^) onto the 40S ribosomal subunit [5]. The eIF2 containing the phosphorylated alpha subunit sequesters eIF2B, which exchanges GDP for GTP on eIF2 for its activation. Sequestration of eIF2B inhibits eIF2-dependent Met-tRNA_i_^Met^ binding to the 40S ribosomal subunit [6, 7]. As a consequence, global translation is repressed by various cellular stresses.

Another well-known example is translational regulation via mammalian target of rapamycin (mTOR). Activation of mTOR leads to the phosphorylation of eIF4E-binding protein 1 (4E-BP1), resulting in the dissociation of 4E-BP1 from the mRNA cap-binding protein eIF4E. Unphosphorylated 4E-BP1 sequesters eIF4E from eIF4F complex through a competitive binding to eIF4E, resulting in the inhibition of cap-dependent translation [8, 9].

The third example of overall translational regulation is the cell cycle-dependent translational fluctuation. M phase is the final stage of the cell cycle wherein a cell divides into two daughter cells. Compared with cells in interphase, cells in M phase show drastic changes in many biological processes, such as inhibition of transcription and translation, chromosome condensation, nuclear envelope breakdown, and assembly of the mitotic spindle [10–12]. It is well established that the translation efficiencies of the most mRNAs are high in interphase but low in M phase [13, 14]. In this study, we investigated the molecular basis of global translational repression during M phase. Cell cycle-dependent translational regulation is required for promoting cell division and survival. For example, the expression of Emi1, an inhibitor of the anaphase-promoting complex (APC), is translationally repressed during mitosis, resulting in the activation of APC during M phase [15]. In contrast, some mRNAs are translationally activated during mitosis. For example, the IRES-dependent translation of Bcl-2 and CDK1 is enhanced during mitosis to prevent cell death caused by the activation of caspases [16].

Several mechanisms have been proposed to explain global translational repression during M phase. One proposed mechanism is through hypo-phosphorylation of eIF4E-binding protein 1 (4E-BP) during mitosis [17] since hypo-phosphorylated 4E-BP1 inhibits cap-dependent translation by interfering with the association of eIF4G to the cap-binding protein, eIF4E [18]. However, it was reported that 4E-BP1 is phosphorylated during mitosis directly by CDK1 [19] or indirectly through activation of the mTORC1 pathway by phosphorylation of the mTORC1-associated protein, raptor [20, 21]. Recently, the involvement of CDK1 in global translational repression was suggested by Dobrikov et al. [22]. The authors suggested that CDK1/cyclin B-dependent phosphorylation of eIF4G1 represses global translation during M phase by enhancing the interaction of eIF4A with eIF4G1. This enhanced interaction decreases the RNA-binding ability of the eIF4G1/eIF4A complex [22]. PKR, a stress-responsive kinase activated by double-stranded RNA, was also suggested to mediate translational repression during M phase [23]. However, mitotic translational repression was not fully restored by blocking the phosphorylation of eIF4G1 and eIF2α [22, 23]. These results suggest the existence of an unknown mechanism by which translational repression occurs during M phase.

The cell cycle is a complex process that requires exquisite regulation of gene expression. In eukaryotes, the two major classes of factors—cyclin-dependent kinases (CDKs) and their positive regulator named cyclins—play key roles in controlling numerous changes during cell division. These evolutionarily conserved regulators are essential for managing cell cycle progression in simple unicellular organisms, such as yeasts, to complex multicellular organisms, such as mammals [10]. In humans, there are more than 20 types of CDKs. Among them, CDK1 to CDK11 are known to function stage-specifically during the G1, G1/S, S, G2, and M phases [24]. CDK11, also known as PITSLRE kinase, has three isoforms that show peculiar expression patterns. Hereafter, the largest, mid-sized, and smallest isoforms of CDK11 are designated as CDK11/p110, CDK11/p58, and CDK11/p46, respectively. The CDK11 isoforms have a common C-terminal region containing the kinase domain. The N-terminal regions of CDK11 isoforms differ from each other. CDK11/p110, which is expressed continuously during cell cycle, localizes in the nucleus and regulates RNA transcription and pre-mRNA splicing through its association with RNA polymerase II and cyclin L [25, 26]. CDK11/p58 is translated from the same mRNA as CDK11/p110. However, the translation of CDK11/p58 is directed by an IRES that resides in the coding region of CDK11/p110 using an alternative in-frame AUG codon during M phase [27]. CDK11/p58 is known to facilitate M phase progression by promoting centrosome maturation and bipolar spindle formation [28, 29]. Independent of cell cycle regulation, the CDK11/p58–cyclin D3 (CCND3) complex inhibits androgen receptor activity through phosphorylation [30]. CDK11/p46 is a truncated form of CDK11/p110 and CDK11/p58 that is through proteolytic cleavage by caspase-3 during apoptosis and represses cap-dependent translation by phosphorylating eIF3F [31–35]. Thus far, no specific cyclin partner of CDK11/p46 activity has been found.

eIF3 is the largest translation initiation factor and is composed of 13 non-identical protein subunits that are named eIF3A to eIF3M (in mammals) [36]. eIF3 connects the 40S ribosomal subunit with the translation initiation factors that are associated with mRNAs (e.g., eIF4F complex and PABP) through direct interactions with eIF4G [37, 38] and the 40S ribosomal subunit [39, 40]. Moreover, eIF3 directly interacts with the multi-factor complex of eIF1–eIF2–eIF5 [41]. Through the interactions with translation factors and an mRNA, eIF3 facilitates the formation of the 48S preinitiation complex.

Translation initiation factors are related to cell proliferation and tumorigenesis. For example, activation of eIF4F complex is involved in tumorigenesis [42, 43], and glycosylation of eIF4G promotes cell proliferation [44]. Alterations in eIF3 subunit expression have been reported for many types of cancer cells, such as the overexpression of eIF3A, B, C, H, I, and M, and under-expression of eIF3E and F [45]. These studies imply that the eIF3 subunits have various roles in cell proliferation. The expression of eIF3F is decreased in melanomas and pancreatic cancer. Furthermore, ectopic expression of eIF3F induces apoptosis and reduces protein synthesis, while knockdown of eIF3F enhances cell proliferation rates [46–49]. These results suggest that eIF3F modulates cell proliferation by repressing translation.

In this study, we investigated the role of CDK11/p58, which is M phase-specifically expressed, in global translational repression during mitosis. Overexpression of CDK11/p58 inhibited cap-dependent translation, and knockdown of CDK11 nullified the repression of cap-dependent translation during M phase. We also found that eIF3F is the molecular target of CDK11/p58 that is responsible for M phase-specific translational repression. Phosphorylation of Thr255 and/or Ser258 in eIF3F by CDK11/p58 resulted in global translational repression in M phase. Our findings suggest that the M phase-specific, IRES-dependent production of CDK11/p58 mediates global translational repression during M phase by phosphorylating the crucial translation factor, eIF3.

## Materials and methods

### Construction of plasmids

The plasmid, which was used for generation of the bicistronic reporter mRNA [50], was kindly provided by Dr. Peter Sarnow (Stanford University). To construct plasmids expressing Flag-tagged CDK11/p110, CDK11/p58, and CDK11/p46, different regions of the CDK11 gene were amplified by PCR from a plasmid containing the CDK11/p110 gene, which was kindly provided by Dr. Susana Valente (The Scripps Research Institute). The primers used in the PCR were as follows: CDK11/p110 (forward: 5′-CCCAAGCTTCGATGGGTGATGAAAAGGAC-3′), CDK11/p58 (forward: 5′-CGCAAGCTTATGAGTGAAGATGAAGAAC-3′), CDK11/p46 (forward: 5′-CGCAAGCTTGTGCCCGACTCCCCT-3′), and a common reverse primer (5′-CCCTCTAGATCAGAACTTGAGGCTGAAG-3′). The amplified DNAs were digested with HindIII and XbaI and then ligated into the HindIII–XbaI site of pcDNA3.1-Flag. The plasmid containing the kinase-dead mutant, CDK11/p58M, was generated by site-directed mutagenesis by mutating Asp224 to Asn in CDK11/p58 [51] using the following primers: forward (5′-CCGGCATCCTCAAGGTGGGTAACTTCGGGCTGGCGCGGGAG-3′) and reverse (5′-CTCCCGCGCCAGCCCGAAGTTACCCACCTTGAGGATGCCGG-3′).

Constructions of the plasmids, pcDNA3.1-Flag and pcDNA3.1-myc, were described previously [52]. To construct plasmids encoding for eIF3F and CCND3 (NM_003754 and NM_001760), the *eIF3F* and *CCND3* genes were amplified by PCR using a human cDNA library (Clontech) with the following primers: eIF3F (forward: 5′-CCGGATCCATGGCCACACCGGCG-3′ and reverse: 5′-CCTCTAGATCACAGGTTTACAAGTTTTTC-3′); CCND3 (forward: 5′-CCAAGCTTATGGAGCTGCTGTG-3′ and reverse: 5′-CCGCGGCCGCCTACAGGTGTATGGCTG-3′). The amplified *eIF3F* DNA was digested with BamHI and XbaI and ligated into the BamHI–XbaI site of pcDNA3.1-Flag or pcDNA3.1-myc. The amplified *CCND3* DNA was digested with HindIII and NotI and ligated into the HindIII–NotI site of pcDNA3.1-Flag. Plasmids encoding for the phosphomimetic and unphosphorylatable eIF3F constructs were generated by site-directed mutagenesis (Fig. 5a). To generate pcDNA3.1-Flag-eIF3F(D1D2) and pcDNA3.1-Flag-eIF3F(A1A2), S46 and T119 in pcDNA3.1-Flag-eIF3F were mutated consecutively. pcDNA3.1-Flag-eIF3F_S46D and pcDNA3.1-Flag-eIF3F_S46A were generated by PCR mutagenesis using pcDNA3.1-Flag-eIF3F with the following primers: eIF3F_S46D (forward: 5′-CCCGCTGCGGCTCCAGCCTCAGACTCAGACCCTGCGGCAGCAGCG-3′ and reverse: 5′-CGCTGCTGCCGCAGGGTCTGAGTCTGAGGCTGGAGCCGCAGCGGG-3′) and eIF3F_S46A (forward: 5′-CCCGCTGCGGCTCCAGCCTCAGCCTCAGACCCTGCGGCAGCAGCG-3′ and reverse: 5′-CGCTGCTGCCGCAGGGTCTGAGGCTGAGGCTGGAGCCGCAGCGGG-3′). pcDNA3.1-Flag-eIF3F(D1D2) and pcDNA3.1-Flag-eIF3F(A1A2) were generated by PCR mutagenesis using pcDNA3.1-Flag-eIF3F_S46D and pcDNA3.1-Flag-eIF3F_S46A, respectively, with the following primers: eIF3F(D1D2) (forward: 5′-GGTGCTGCCCGAGTTATCGGGGACCTGTTGGGAACTGTCGACAAA-3′ and reverse: 5′-TTTGTCGACAGTTCCCAACAGGTCCCCGATAACTCGGGCAGCACC-3′) and eIF3F(A1A2) (forward: 5′-GGTGCTGCCCGAGTTATCGGGGCCCTGTTGGGAACTGTCGACAAA-3′ and reverse: 5′-TTTGTCGACAGTTCCCAACAGGGCCCCGATAACTCGGGCAGCACC-3′). pcDNA3.1-Flag-eIF3F(D3D4) and pcDNA3.1-Flag-eIF3F(A3A4) were generated by PCR mutagenesis using pcDNA3.1-Flag-eIF3F with the following primers: eIF3F(D3D4) (forward: 5′-GAGTTGACCTGATCATGAAGGACTGCTTTGACCCCAACAGAGTGATTGGACT-3′ and reverse: 5′-AGTCCAATCACTCTGTTGGGGTCAAAGCAGTCCTTCATGATCAGGTCAACTC-3′) and eIF3F(A3A4) (forward: 5′-GAGTTGACCTGATCATGAAGGCCTGCTTTGCCCCCAACAGAGTGATTGGACT-3′ and reverse: 5′-AGTCCAATCACTCTGTTGGGGGCAAAGCAGGCCTTCATGATCAGGTCAACTC-3′).

### Synthetic siRNA

The negative control siControl and siCDK11 were purchased from Bioneer. The sequence of siCDK11 has been previously described [30].

### Establishment of cell lines stably expressing eIF3F variants

HeLa cells were cultivated in Dulbecco’s modified Eagle’s medium (DMEM; Gibco BRL) supplemented with 10% fetal bovine serum (Hyclone) and 1% penicillin/streptomycin (Invitrogen) under 5% CO_2_. To establish cell lines ectopically expressing eIF3F variants (eIF3F(WT), eIF3F(A1A2), or eIF3F(A3A4)), pcDNA3.1-Flag-eIF3F, pcDNA3.1-Flag-eIF3F(A1A2), or pcDNA3.1-Flag-eIF3F(A3A4) were transfected into HeLa cells with Lipofectamine 3000 (Invitrogen) according to the manufacturer’s instruction. Control HeLa cells were transfected with the pcDNA3.1-Flag. Cells containing the plasmids were selected with 200 μg/mL of hygromycin B (AG Scientific).

### In vitro transcription and transfection

To synthesize the 5′-capped, bicistronic reporter mRNA (RCF), in vitro transcription was performed using the T7 RNA polymerase in the presence of the 3′O-Me-m^7^G(5′)ppp(3′)G (anti-reverse cap analog) and the bicistronic reporter plasmids that had been linearized by digestion with XbaI. Transfections of reporter mRNAs, synthetic siRNAs and plasmid DNAs were carried out using Lipofectamine 3000 (Invitrogen) according to the manufacturer’s instruction.

### Measurement of the translation efficiencies of reporter genes

To measure the efficiencies of cap-dependent and CrPV IRES-dependent translation, the indicated effector plasmids were transfected into ~ 40% confluent HeLa cells grown in six-well plates. After 48 h, cells were transfected with the bicistronic reporter mRNAs (RCF) and then incubated for 4 h. The cells were treated with passive lysis buffer (Promega) and harvested. *Renilla* and firefly luciferase activities were measured using a dual-luciferase kit (Promega) according to the manufacturer’s instruction.

### Synchronization of cells

HeLa cells were synchronized at G1/S boundary or in M phase using the double thymidine or thymidine–nocodazole block methods, respectively [53]. Briefly, HeLa cells were cultivated to 40% confluence and then treated with 2 mM thymidine (Sigma) for 16 h. The cells were washed three times with PBS at 37 °C and then cultivated in fresh media for 8 h. For synchronization of cells in interphase, cells were further cultivated in the presence of 2 mM thymidine for 16 h. For synchronization of cells in M phase, cells were further cultivated in the presence of 100 ng/mL nocodazole (Sigma) for 16 h.

Synchrony of cells was confirmed by flow cytometry analysis after staining the cells with propidium iodide. After synchronization, cells were trypsinized, collected by centrifugation, washed twice with cold PBS, and fixed in 70% methanol/distilled water for 1 h at 4 °C. The cells were then precipitated by slow centrifugation, washed with cold PBS, and incubated with 0.2 mg/mL of RNaseA (Sigma) for 1 h at 37 °C. The cells were incubated with 5 μg/mL propidium iodide (BioLegend) for 30 min at RT. Ten thousand cells from each sample were analyzed by flow cytometry using a Canto II instrument (Becton Dickinson) to determine DNA content as an indicator of synchrony.

### SUnSET

HeLa cells were transfected with the indicated plasmids and cultivated on plates. After transfection, cells were synchronized in interphase or M phase as described above. After synchronization, cells were washed three times with PBS at 37 °C and cultivated in growth medium in the presence of 5 mM puromycin (Sigma) for 15 min. Newly synthesized proteins were detected by western blotting using an anti-puromycin antibody.

### Immunoprecipitation, phos-tag PAGE, and western blotting

HeLa cells were transfected with the indicated plasmids and cultivated on plates. At 48 h after transfection, cells were washed with cold PBS and harvested with ice-cold lysis buffer (0.1% NP-40, 40 mM HEPES–KOH (pH 7.5), 100 mM KCl, 1 mM EDTA, 10 mM NaF, 10 mM β-glycerophosphate, 2 mM Na_3_VO_4_, and 1 mM PMSF). The cells were lysed by sonication, and cell debris was cleared by centrifugation. For immunoprecipitation, 300 μg of whole-cell extracts (WCEs) were incubated with 1 μg of anti-myc antibody (GeneTex, GTX29106) and 10 μL of Protein A agarose (Roche) at 4 °C for 2 h with constant rotation. The beads were collected by centrifugation and washed four times with fresh lysis buffer. WCEs and precipitates were resolved by SDS-PAGE and then transferred to PVDF membrane (Millipore). To separate phosphorylated from unphosphorylated proteins, Phos-tag (Wako; 50 μM) and MnCl_2_ (50 μM) were added to the polyacrylamide gel [54]. The resolved proteins were analyzed by western blotting using the relevant antibodies. Band quantification was performed with Image J software [55]. The following primary antibodies were used for western blotting: control rabbit IgG (Santa Cruz, sc-2027), anti-Flag (Sigma, F1804), anti-Myc (GeneTex, GTX29106), anti-eIF3F (Rockland, 600-401-934), anti-CDK11 (Abcam, ab19393), anti-puromycin (Millipore, MABE343), and anti-GAPDH (AbD Serotec, 4699-9555).

### Statistical analysis

All the statistical analyses were performed with GraphPad Prism 8.0. Data are expressed as means ± standard deviations. To determine statistical significance, Student’s *t* tests (two-tailed) were performed and *p* values $\le$ 0.05 were considered statistically significant.

## Results

### Mitotic phase-specific kinase, CDK11/p58, represses cap-dependent translation

**Fig. 1:**
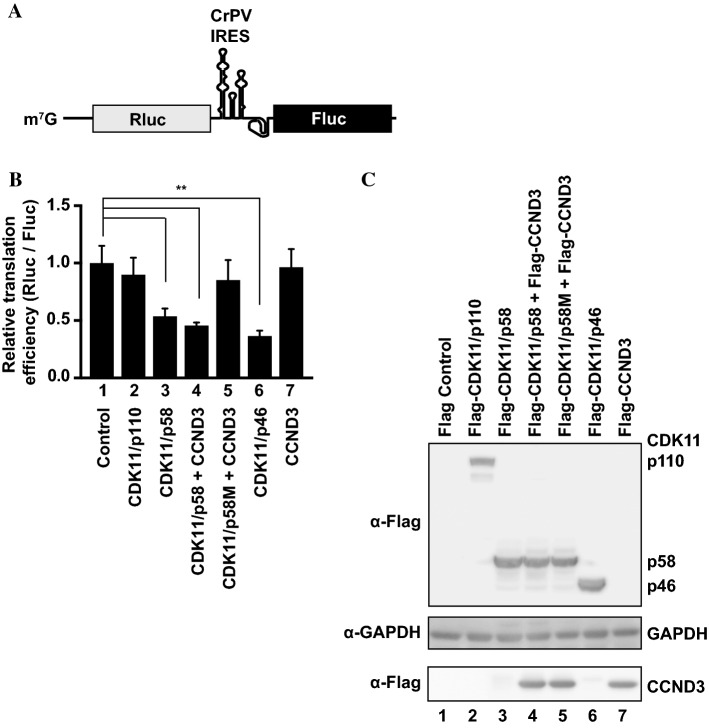
CDK11/p58 represses cap-dependent translation. **a** Schematic diagram of a bicistronic reporter mRNA (RCF) containing the cap structure (m^7^G) at the 5′ end, *Renilla* luciferase (Rluc) gene at the first cistron, cricket paralysis viral (CrPV) IRES at the intercistronic region, and firefly luciferase (Fluc) gene at the second cistron. **b** Cap- and CrPV IRES-dependent translation of the reporter mRNA (RCF) in cells ectopically expressing CDK11 isoforms were monitored by measuring *Renilla* and firefly luciferase activities. At 48 h post-transfection of the corresponding plasmids encoding for effector CDK11 isoforms, HeLa cells were transfected with the bicistronic reporter RNA (RCF) that was synthesized by in vitro transcription. After 4 h, cells were lysed, and luciferase activities were measured. The relative cap-dependent translation efficiencies, which were normalized to the CrPV IRES-dependent translation activity in each sample, are depicted. The cap-dependent translation activity in the control vector-transfected cells was set to 1 (lane 1). Experiments were repeated three times with duplicate samples. The columns and bars represent the means and ± standard deviations, respectively. Asterisks (**) depict *P* < 0.005, lanes 3, 4, and 6 compared with lane 1. **c** The levels of ectopically expressed proteins (CDK11 isoforms and CCND3) in cells were monitored by western blot using an anti-Flag antibody. GAPDH levels were also monitored by western blot using an anti-GAPDH antibody as an endogenous protein control

We investigated whether various CDK11 isoforms can modulate translation efficiency. To test this, we transfected HeLa cells with plasmids encoding three different isoforms of CDK11 (p110, p58, and p46) (Fig. 1) and monitored cap-dependent and IRES-dependent translation of a reporter mRNA. We used a bicistronic mRNA (named RCF) containing CrPV IRES, which does not require a translation initiation factor for IRES-mediated translation [50], at the intercistronic region to direct the translation of the firefly luciferase (Fluc) gene (Fig. 1a). In contrast, translation of the *Renilla* luciferase (Rluc) gene in the first cistron occurs in a cap-dependent manner. Thus, relative luciferase activity of Rluc to Fluc represents cap-dependent translation efficiency. Expression of CDK11/p58 and co-expression of CDK11/p58 and CCND3 significantly repressed cap-dependent translation, while expression of CCND3 alone showed no effect (Fig. 1b, lanes 3, 4, and 7). In contrast, CrPV IRES-dependent translation was not affected by the expression of CDK11/p58 or co-expression of CDK11/p58 and CCND3.

To examine whether kinase activity of CDK11/p58 is required for repressing cap-dependent translation, we monitored the effect of ectopic expression of CDK11/p58M—a kinase-dead mutant of CDK11/p58—on cap-dependent translation [51]. CDK11/p58M showed no effect on cap-dependent translation (Fig. 1b, lane 5). As reported previously [31, 32], CDK11/p46, the apoptosis-specific isoform of CDK11, repressed cap-dependent translation (Fig. 1b, lane 6). In contrast, ectopic expression of CDK11/p110, which is continuously expressed during cell cycle, showed no effect on cap-dependent translation (Fig. 1b, lane 2). These results indicate that CDK11/p58, which is expressed during M phase, inhibits cap-dependent translation, and that its kinase activity is required for translational repression. The levels of ectopically expressed CDK isoforms and CCND3 were monitored by western blotting (Fig. 1c).

### CDK11 is required for repressing global translation during mitosis

**Fig. 2:**
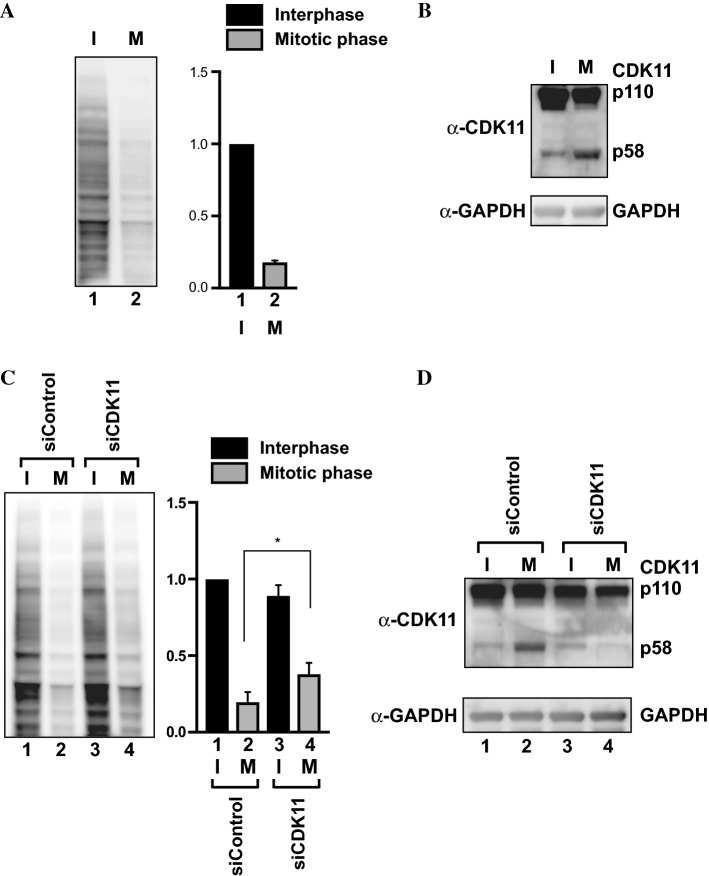
Translation repression in M phase is nullified by knockdown of CDK11. **a** Global translation in interphase or M phase-arrested cells was monitored by western blotting using SUnSET (left panel). Synchronization of cells in either interphase or M phase was achieved as described in “Materials and methods”. Puromycin was treated for 15 min after synchronization and the puromycin incorporation into newly synthesizing proteins was detected by western blotting using SUnSET. The band densities of newly synthesized proteins in interphase and M phase were measured and normalized to the amounts of total proteins measured by Coomassie blue staining (Fig. S1b). Relative values are depicted on the right panel. The sum of the band intensities from cells in interphase was set to 1 (lane 1). Experiments were repeated three times. Values are represented as the means and ± standard deviations, respectively. **b** Protein levels of CDK11/p110 and CDK11/p58 in interphase- and M phase-synchronized cells were monitored by western blotting using an anti-CDK11 antibody. **c** The effect of CDK11 knockdown on cell cycle-dependent translation. HeLa cells were transfected with either a control siRNA (lanes 1 and 2) or a siRNA against CDK11 (lanes 3 and 4) 3 h before synchronization. Translation efficiencies were measured as in **a**. Band densities of newly synthesized proteins in interphase and M phase were measured; relative values are depicted on the right panel. The sum of band intensities from cells in interphase treated with a control siRNA was set to 1 (lane 1). Experiments were repeated three times. The columns and bars represent the means and ± standard deviations, respectively. Asterisk (*) depicts *P* < 0.05, lane 4 compared with lane 2. **d** Protein levels of CDK11/p110 and CDK11/p58 in interphase- and M phase-synchronized cells treated with either a control siRNA (lanes 1 and 2) or an siRNA against CDK11 (lanes 3 and 4), were monitored by western blotting using an anti-CDK11 antibody

We examined global translation during interphase and M phase by synchronizing HeLa cells in interphase (early S phase) by double thymidine block, or in M phase by thymidine–nocodazole block. Cell cycle synchronization was confirmed by a flow cytometry method (Fig. S1a). After synchronization of cells, we measured global translation rates by the surface sensing of translation (SUnSET) method. SUnSET allows the detection of newly synthesized proteins using puromycin, which is incorporated into elongating polypeptide chains. Newly synthesized proteins can be detected by western blotting using an anti-puromycin antibody [56]. To compare global translation rates, the band intensity of newly synthesized, puromycin-labeled proteins visualized by western blotting was normalized to the amount of total proteins visualized by Coomassie blue staining (Figs. 2a and S1b).

Global translation of cells synchronized in M phase was decreased by about 80% compared with cells synchronized in interphase (Fig. 2a). A previous study, which measured mitotic translation using SUnSET, showed similar repression patterns [22]. The global repression of translation that occurred in this study was likely attributed to two distinct mechanisms. Approximately half of the reduction is likely attributed to cells being in M phase, while the other half is likely attributed to the adverse effect of the microtubule inhibitor used during cell synchronization [15] (for details, refer to the discussion section).

As expected, the levels of CDK11/p58 were increased in cells arrested in M phase (Fig. 2b). To investigate the role of CDK11 in mediating global translation during mitosis, we transfected siRNAs against CDK11 into HeLa cells and then synchronized cells at interphase or M phase (Fig. S2a). The global translation in the synchronized cells was monitored by the SUnSET method (Figs. 2c and S2b). Knockdown of CDK11 almost completely abolished M phase-specific expression of CDK11/p58 but partially reduced the level of CDK11/p110 (Fig. 2d). The discrepancy between levels of CDK11/p58 and CDK11/p110 after treatment of siRNAs targeting the same mRNA is likely attributed to the stability of these isoforms. It is noteworthy that CDK11/p58 exists only in M phase, indicating its labile character. In contrast, CDK11/p110 remains constant across the cell cycle, and thus, may not require fast degradation. The repression of global translation during mitosis was partially nullified when CDK11/p58 was depleted (Fig. 2c, compare lane 2 with 4). In contrast, translation of cells in interphase was not affected by CDK11/p58 depletion (Fig. 2c, compare lane 1 with 3). These results indicate that CDK11/p58 is required for global translational repression during mitosis since CDK11/p110 does not affect translation (Fig. 1b, lane 2).

### CDK11/p58 interacts with eIF3F

**Fig. 3:**
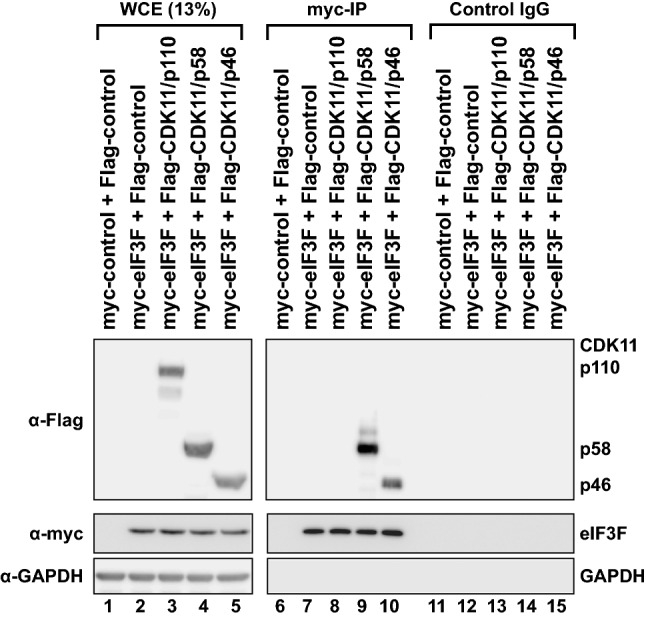
CDK11/p58 interacts with eIF3F. Flag-CDK11 isoforms (p110, p58, and p46) and myc-eIF3F were co-transfected to HeLa cells. The myc-eIF3F was immunoprecipitated using an anti-myc antibody-conjugated protein A agarose resin (lanes 6–10). Non-specific interactions were monitored using an anti-rabbit antibody-conjugated protein A agarose resin (control, lanes 11–15). Protein levels of CDK11 isoforms and eIF3F were monitored by western blotting using anti-Flag and anti-myc antibodies, respectively. GAPDH levels were monitored by western blotting using an anti-GAPDH antibody as an endogenous protein control

To explore how CDK11/p58 affects translation, we examined whether a substrate of CDK11/p58 is involved in translational regulation. Previous studies revealed that CDK11/p46, the apoptosis-specific CDK11 isoform, directly interacts with and phosphorylates eIF3F to repress translation [31, 32]. Thus, we examined whether other CDK11 isoforms can also interact with eIF3F by co-immunoprecipitation (Fig. 3). As expected, eIF3F co-precipitated CDK11/p46 (Fig. 3, lane 10). Interestingly, eIF3F also co-precipitated CDK11/p58, but not CDK11/p110 (Fig. 3, lanes 9 and 8). These results suggest that CDK11/p58 may phosphorylate eIF3F and affect translational regulation similar to CDK11/p46.

### eIF3F is phosphorylated by CDK11/p58 in mitotic phase

**Fig. 4:**
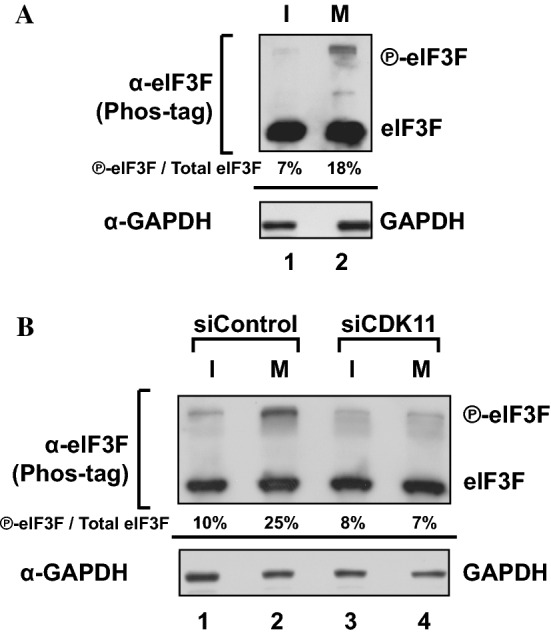
Endogenous eIF3F is phosphorylated by CDK11/p58 during M phase. **a** The phosphorylation levels of endogenous eIF3F in interphase- and M phase-synchronized HeLa cells were measured by western blotting using Phos-tag SDS-PAGE. Slower migration of phosphorylated eIF3F in Phos-tag SDS-PAGE results in separation of fast-migrating under-phosphorylated and slow-migrating hyper-phosphorylated eIF3F (  -eIF3F) bands in the gel. Protein levels of  -eIF3F and eIF3F were detected by western blotting using an anti-eIF3F antibody. The ratios of  -eIF3F/total eIF3F are depicted. The levels of GAPDH were monitored as a loading control. **b** Phosphorylation levels of endogenous eIF3F in interphase- or M phase-synchronized HeLa cells with (lanes 3 and 4) or without knockdown (lanes 1 and 2) of CDK11 were monitored by western blotting using Phos-tag SDS-PAGE. Protein levels of  -eIF3F and eIF3F were detected by western blotting using an anti-eIF3F antibody. The ratios of  -eIF3F/total eIF3F are depicted. The levels of GAPDH were monitored as a loading control

Previous studies showed that eIF3F is phosphorylated during apoptosis by CDK11/p46 when cap-dependent translation is repressed [31, 32]. We examined the phosphorylation levels of endogenous eIF3F during interphase and M phase using Phos-tag—a method that can monitor the phosphorylation status of a protein by SDS-PAGE [54]. Phosphorylation of eIF3F in HeLa cells was increased by approximately 2.5-fold during M phase (Fig. 4).

Since CDK11/p58 is specifically expressed during mitosis (Fig. 2b) and interacts with eIF3F (Fig. 3), we investigated whether CDK11/p58 is responsible for the M phase-specific phosphorylation of eIF3F. Knockdown of CDK11 drastically reduced the level of phosphorylated eIF3F in M phase cells to that in interphase cells (Fig. 4b, compare lane 4 with 2 and 3). The result indicates that CDK11/p58 is responsible for the M phase-specific phosphorylation of eIF3F. However, we cannot completely rule out the possibility of indirect phosphorylation of eIF3F by CDK11/p58 via an unidentified kinase activated by CDK11/p58 even though the indirect phosphorylation of eIF3F is less likely since CDK11/p58 associates with eIF3F (Fig. 3). It is worth to note that 7–10% of eIF3F proteins are phosphorylated in the control interphase cells and that similar amounts of eIF3F proteins are phosphorylated in both interphase and M phase of CDK11 knockdown cells (Fig. 4). The results suggest that the hyper-phosphorylation of eIF3F in M phase is solely attributed to CDK11/p58 and that the basal phosphorylation of eIF3F, which remains at the same level across the cell cycle in CDK11 knockdown cells (Fig. 4b), is likely executed by an unknown kinase(s) other than CDK11.

### Phosphorylation of eIF3F represses cap-dependent translation

**Fig. 5:**
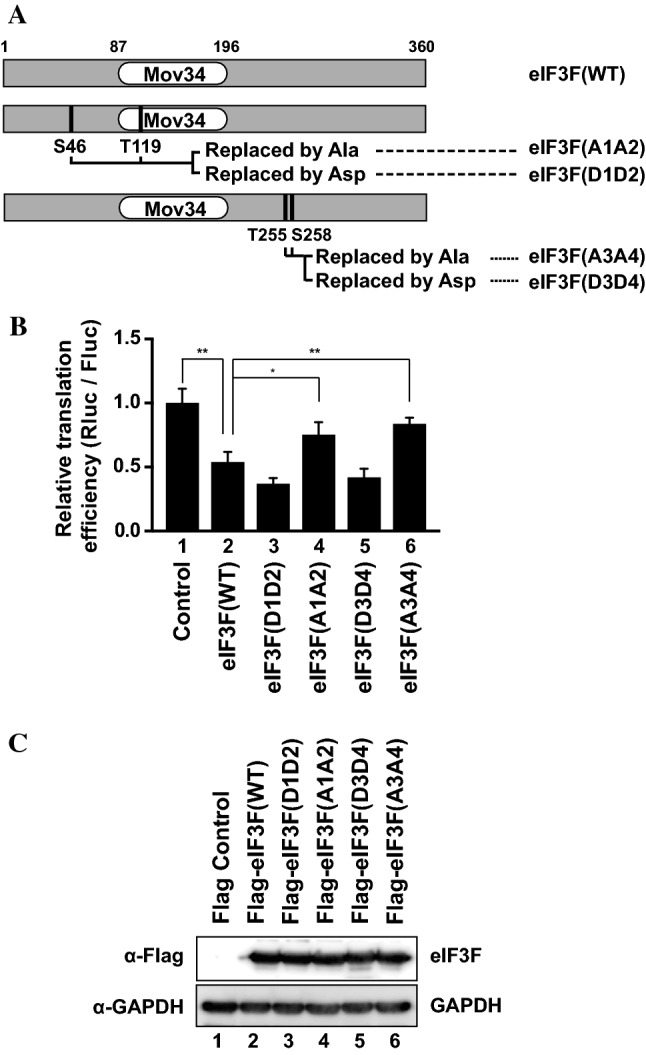
Unphosphorylatable mutants of eIF3F do not repress translation. **a** Schematic diagrams of phospho-mimetic mutants [eIF3F(D1D2) and eIF3F(D3D4)], and unphosphorylatable mutants [eIF3F(A1A2) and eIF3F(A3A4)] of eIF3F. **b** The effects of ectopic expression of eIF3F(WT) and its derivatives on cap-dependent translation were monitored as described in Fig. 1 except that effector plasmids encoding eIF3F derivatives instead of CDK11 isoforms were used. The relative cap-dependent translation efficiencies, which were normalized to CrPV IRES-dependent translation activity in each sample, are depicted. The cap-dependent translation activity in control vector-transfected cells was set to 1 (lane 1). Experiments were repeated three times with duplicate samples. The columns and bars represent the means and ± standard deviations, respectively. Asterisk (*) depicts *P* < 0.05, lane 4 compared with lane 2. Asterisks (**) depict *P* < 0.005, lane 6 compared with lane 2. **c** The levels of ectopically expressed proteins (eIF3F and its derivatives) in cells were monitored by western blotting using an anti-Flag antibody. The level of GAPDH protein was also monitored using an anti-GAPDH antibody as an endogenous protein control

A previous study revealed that Ser46 and Thr119 are the CDK11/p46-directed phosphorylation sites in eIF3F [32]. However, we speculated that CDK11/p58 might phosphorylate different residues since substrate specificity of CDKs is often determined by the cyclin associated with the particular CDK [57]. A proteome analysis of cell cycle-dependent phosphorylation revealed that two residues in eIF3F (Thr255 and Ser258) are specifically phosphorylated during M phase even though the kinase responsible for their phosphorylation is unknown [58]. We tested whether CDK11/p58, an M phase-specific kinase, is responsible for the phosphorylation of these residues of eIF3F. For this purpose, we constructed four eIF3F mutants [eIF3F(A1A2), eIF3F(D1D2), eIF3F(A3A4), and eIF3F(D3D4)] (Fig. 5a): eIF3F(A1A2) contains substitution mutations from Ser46 and Thr119 to unphosphorylatable alanines (S46A and T119A); eIF3F(D1D2) contains substitution mutations from Ser46 and Thr119 to phospho-mimetic aspartic acids (S46D and T119D); eIF3F(A3A4) contains substitution mutations from Thr255 and Ser258 to unphosphorylatable alanines (T255A and S258A); eIF3F(D3D4) contains substitution mutations from Thr255 and Ser258 to phospho-mimetic aspartic acids (T255D and S258D).

Wild-type eIF3F [eIF3F(WT)] and eIF3F mutants (Fig. 5a) were transfected into HeLa cells and expressed at similar levels (Fig. 5c). Cap- and CrPV IRES-dependent translation in these cells were monitored using the CrPV bicistronic reporter mRNA, RCF (Fig. 1a). Expression of wild-type eIF3F reduced cap-dependent translation by 40% (Fig. 5b, compare lane 1 with 2). Expression of the phospho-mimetic mutants of eIF3Fs [eIF3F(D1D2) and eIF3F(D3D4)] further reduced cap-dependent translation (Fig. 5b, compare lane 2 with 3 and 5). In contrast, expression of the unphosphorylatable eIF3F mutants [eIF3F(A1A2) and eIF3F(A3A4)] partially nullified the translation repression by eIF3F (Fig. 5b, compare lane 2 with 4 and 6). These data indicate that phosphorylation of Ser46, Thr119, Thr255, and/or Ser258 in eIF3F is involved in repressing cap-dependent translation. CrPV IRES-dependent translation was not affected by the ectopic expression of eIF3F variants.

### CDK11/p58 phosphorylates Thr255 and/or Ser258 of eIF3F

**Fig. 6:**
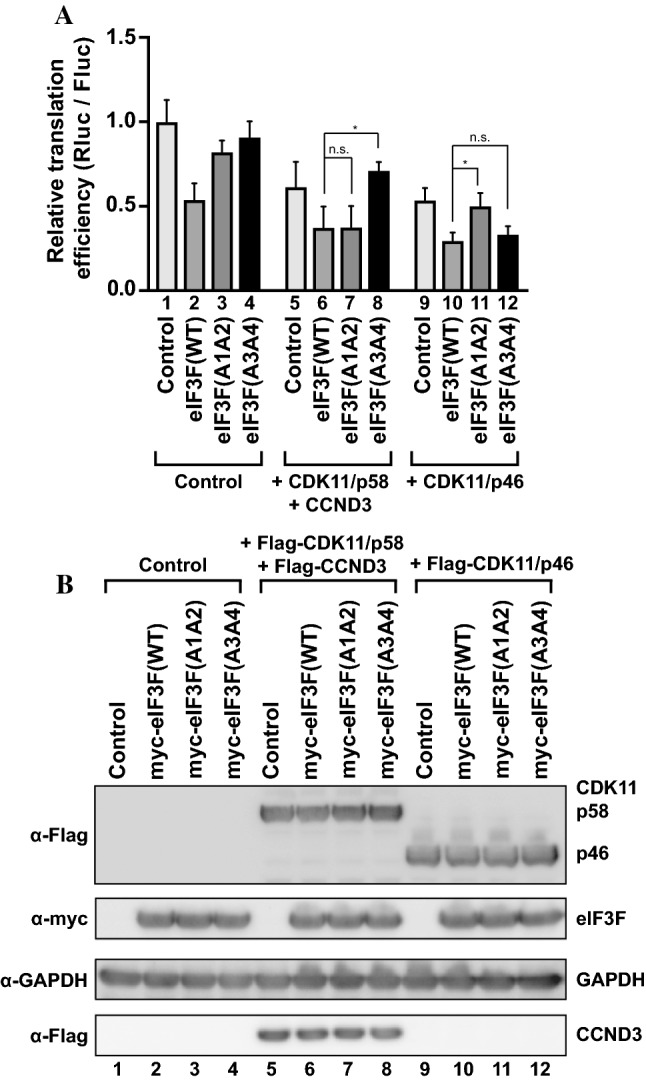
CDK11/p58 phosphorylates Thr255 and/or Ser258 on eIF3F. **a** eIF3F(A3A4) nullifies the cap-dependent translation repression by CDK11/p58, but eIF3F(A1A2) nullifies the cap-dependent translation repression by CDK11/p46. Plasmids encoding Flag-CDK11/p46, Flag-CDK11/p58 plus Flag-CCND3, myc-eIF3F, or its derivatives were co-transfected into HeLa cells as indicated. The effects of co-expression of eIF3F derivatives and CDK11 isoforms on cap-dependent translation were monitored as described in Fig. 1, except that the effector plasmids encoding both eIF3F derivatives and CDK11 isoforms were used. The relative cap-dependent translation efficiencies, which were normalized to CrPV IRES-dependent translation activity in each sample, are depicted. The cap-dependent translation activity in control vector-transfected cells was set to 1 (lane 1). Experiments were repeated three times with duplicate samples. The columns and bars represent the means and ± standard deviations, respectively. Asterisk (*) depicts *P* < 0.05, lane 8 compared with lane 6, and lane 11 compared with lane 10; *n.s.* non-significant (*P* > 0.05). **b** The levels of ectopically expressed proteins (CDK11 isoforms, eIF3F derivatives, and CCND3) in cells were monitored by western blotting using anti-Flag and anti-myc antibodies. GAPDH levels were monitored using an anti-GAPDH antibody as an endogenous protein control

To investigate which amino acid residues are phosphorylated by CDK11/p58, we co-transfected the plasmids expressing eIF3F variants with those expressing CDK isoforms and then monitored cap- and IRES-dependent translation using the CrPV bicistronic reporter mRNA (Fig. 6). As shown in Fig. 1b, expression of CDK11/p58 together with CCND3 reduced cap-dependent translation (Fig. 6a, compare lane 1 with 5). Similarly, co-expression of wild-type eIF3F and CDK11/p58 together with CCND3 further reduced cap-dependent translation (Fig. 6a, compare lane 5 with 6). Co-expression of eIF3F(A1A2) and CDK11/p58 together with CCND3 reduced cap-dependent translation to the same level as wild-type eIF3F (Fig. 6a, compare lane 5 with 6 and 7). The results indicate that eIF3F(A1A2) was phosphorylated by CDK11/p58, which demonstrates that Ser46 and Thr119 of eIF3F are not targeted by CDK11/p58. In contrast, co-expression of eIF3F(A3A4) and CDK11/p58 together with CCND3 did not further reduce cap-dependent translation from the level observed by transfection of CDK11/p58 with CCND3 (Fig. 6a, compare lane 5 with 8). Thus, phosphorylation of eIF3F(A3A4) by CDK11/p58 did not occur, which demonstrates that Thr255 and/or Ser258 of eIF3F are the target sites for CDK11/p58.

As a control, we performed co-expression experiments with CDK11/p46. As previously reported [32], co-expression of eIF3F(A1A2) and CDK11/p46 did not further reduce cap-dependent translation from the expression observed with CDK11/p46 alone (Fig. 6a, compare lane 9 with 11). This indicates that CDK11/p46 did not phosphorylate eIF3F(A1A2), which demonstrates that Ser46 and Thr119 of eIF3F are the target sites for CDK11/p46. In contrast, co-expression of eIF3F(A3A4) and CDK11/p46 reduced cap-dependent translation to the same level as wild-type eIF3F (Fig. 6a, compare lane 9 with 10 and 12). Thus, eIF3F(A3A4) was phosphorylated by CDK11/p46, demonstrating that Thr255 and Ser258 are not targeted by CDK11/p46. These data are consistent with the previous report [32]. Taken together our results suggest that CDK11/p58 represses cap-dependent translation by phosphorylating the M phase-specific phosphorylation sites (Thr255 and/or Ser258) of eIF3F, and that the CDK11/p58- and CDK11/p46-directed phosphorylation sites differ from each other.

We investigated whether the CDK11/p58-dependent phosphorylation of eIF3F affects eIF3 complex formation using a co-immunoprecipitation method. We performed co-immunoprecipitation experiments after ectopic expressions of two components of the eIF3 complex (Fig. S5a–c). Alternatively, we performed co-immunoprecipitation experiments after ectopic expression of one of the eIF3F variants to monitor eIF3 complex formation with the endogenous components of eIF3 (Fig. S5d). The results indicate that both phosphorylated [reflected by phospho-mimetic mutants eIF3F(D3D4)] and unphosphorylated eIF3F [reflected by unphosphorylatable mutants eIF3F(A3A4)] are incorporated into the eIF3 complex to the same extent. It is noteworthy that eIF3B, which does not directly interact with eIF3F in the eIF3 complex [59], was also co-precipitated with eIF3F variants to the same extent (Figs. S5a and S5d). Therefore, we conclude that the effect of eIF3F phosphorylation on translation is not attributed to changes in the efficiency of eIF3 complex formation.

To further investigate the presence of phosphorylated eIF3F in the eIF3 complex, we performed sucrose gradient analyses with HeLa cells synchronized at the interphase or mitotic phase (Fig. S6). As expected, eIF3B and eIF3F were mainly enriched in two fractions where eIF3 complex (fraction A) and the free forms of eIF3 components (fraction D) migrated during centrifugation (Fig. S6a). We analyzed the phosphorylated form of eIF3F in fractions A–D using Phos-tag SDS-PAGE. To our surprise, the phosphorylated eIF3F was not enriched in either fraction A or D. Instead, the phosphorylated eIF3F was enriched in fraction B having intermediate sedimentation velocity between the eIF3 complex and free eIF3F (Fig. S6c). This may indicate that the phosphorylated eIF3F composes an immature eIF3 complex which is lighter than the complete eIF3 complex (see below).

### Expression of the unphosphorylatable eIF3F(A3A4) mutant nullifies M phase-specific translational repression

**Fig. 7:**
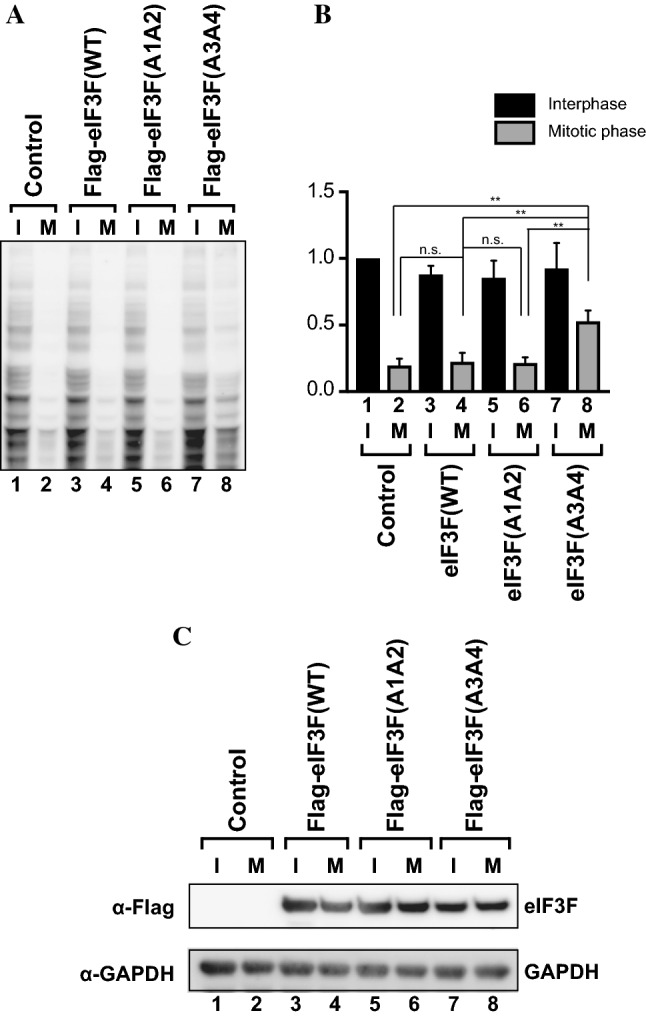
Phosphorylation of Thr255 and/or Ser258 in eIF3F is required for M phase-specific translational repression. HeLa cell lines ectopically expressing eIF3F(WT), eIF3F(A1A2), or eIF3F(A3A4) were established as described in “Materials and methods”. **a** Global translation of the established cells, which were synchronized in interphase (I) or M phase (M), was monitored by western blotting using the SUnSET method. **b** Band densities of newly synthesized proteins in panel (**a**) were measured and normalized to the amount of total proteins (Fig. S3b). The relative values are depicted as the sum of normalized band intensities in interphase cells established with a control vector is set to 1. Experiments were repeated three times. The columns and bars represent the means and ± standard deviations, respectively. **c** The levels of Flag-eIF3F(WT), Flag-eIF3F(A1A2), and Flag-eIF3F(A3A4) in the established cells in interphase- and M phase-synchronized cells were monitored by western blotting using an anti-Flag antibody. GAPDH levels were monitored using an anti-GAPDH antibody as an endogenous protein control

**Fig. 8:**
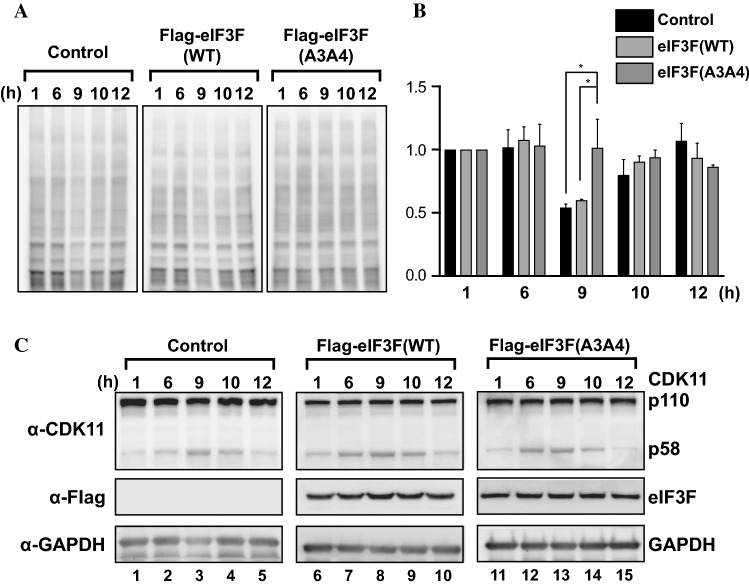
Unphosphorylatable eIF3F(A3A4) completely nullifies M phase-specific translational repression. **a** HeLa cells ectopically expressing eIF3F variants were synchronized at G1/S boundary by double thymidine block and resumed cell cycle progression by removing the compound. Cells were harvested at the indicated time points after cell cycle resumption, and the global translation activity of cells was monitored by western blotting using the SUnSET method. **b** Band densities of newly synthesized proteins in **a** were measured and normalized to the amount of total proteins (Fig. S4b). The relative values are depicted as the sum of normalized band intensities of each cell line at 1 h after cell cycle resumption is set to 1. Experiments were repeated three times. The columns and bars represent the means and ± standard deviations, respectively. **c** The levels of CDK11/p110, CDK11/p58, Flag-eIF3F(WT), and Flag-eIF3F(A3A4) in the established cells were monitored by western blotting using anti-CDK11 and anti-Flag antibodies. GAPDH levels were monitored using an anti-GAPDH antibody as an endogenous protein control

We further investigated the effects of eIF3F phosphorylation by CDK11/p58 on M phase translation using newly established HeLa cell lines ectopically expressing either wild-type eIF3F [eIF3F(WT)] or unphosphorylatable eIF3F mutants [eIF3F(A1A2) and eIF3F(A3A4)] (Figs. 7, 8). We synchronized the HeLa cells expressing eIF3F variants at interphase or M phase by chemical treatments. The synchronization of cells was monitored by flow cytometry (Fig. S3a). The global translation level of the cells in interphase and M phase was monitored by western blotting using the SUnSET method (Fig. 7a) and normalized to the total protein level in the cell extracts (Figs. 7b and S3c). Translation levels of the cells in interphase were similar irrespective of the expression of wild-type eIF3F or unphosphorylatable mutant eIF3Fs (Fig. 7b, compare lanes 1, 3, 5 and 7). Unlike the cells transiently expressing eIF3F variants, wherein cap-dependent translation was repressed by the ectopic expression of eIF3 variants (Fig. 5), the stable cell lines expressing eIF3F variants did not show apparent inhibition of global protein synthesis during interphase. This is likely because the modulation of translation by eIF3F variants during interphase is already reflected in the amounts of total proteins.

A striking difference in global translation was observed in M phase-arrested cells expressing eIF3F(A3A4), which cannot be phosphorylated by CDK11/p58 (Fig. 7a, compare lanes 2, 4, 6, and 8). The level of overall translation in cells in M phase expressing eIF3F(A3A4) was twofold higher than that in M phase cells expressing wild-type eIF3F or eIF3F(A1A2) (Fig. 7b, compare lane 8 with 4 and 6).

Even though translational repression in M phase cells, synchronized by nocodazole treatment, was partially nullified by the expression of eIF3F(A3A4), nocodazole induces stress-related translational inhibition that obscures the magnitude of translation repression in M phase [60]. Therefore, we explored an alternative approach to investigate the M phase-specific translational repression mediated by the phosphorylation of eIF3F under normal conditions. To minimize the external stresses during M phase, we synchronized cells at G1/S boundary by double thymidine block and resumed cell cycle progression by removing the compound as described previously [53]. The amount of DNA per cell was monitored by flow cytometry at various time points after the resumption of cell cycle progression (Fig. S4a). Most cells were in G2 phase at 6 h after the cell cycle resumption, and the majority of cells were in M phase at 9 h after the cell cycle resumption. Most cells were in G1 phase at 10 and 12 h after the cell cycle resumption (Fig. S4a). As expected, the level of CDK11/p58 was the highest in cells at 9 h after the cell cycle resumption when the population of M phase cell is the highest (Fig. 8c).

We monitored the global translation levels of control, eIF3F(WT)-expressing, and eIF3F(A3A4)-expressing cells at various time points after cell cycle resumption by western blotting using the SUnSET method (Fig. 8a). The amounts of newly synthesized proteins at each time point were normalized to the amounts of total proteins measured by Coomassie blue staining (Fig. S4b), and the relative translational efficiencies at various time points after cell cycle resumption are depicted as a graph (Fig. 8b). The global translation level of M phase cells decreased to about 50% compared with interphase cells in control and eIF3F(WT)-expressing cells. In contrast, the global translation level of M phase cells remained the same as interphase cells in eIF3F(A3A4)-expressing cells (Fig. 8b). The data indicate that translation is globally repressed about 50% in M phase cells and that phosphorylation of Thr255 and/or Ser258 in eIF3F by CDK11/p58 plays a major role in the M phase-specific translational repression.

## Discussion

Conventional studies on cell cycle-dependent translational regulation [13, 14] and genome-wide analysis of mRNAs that are translated at specific cell cycle phases revealed that cap-dependent translation generally decreases in M phase, while translation of some mRNAs continues, or even increases, in M phase [15]. In this study, we sought to understand the molecular basis of M phase-specific translational inhibition. We found that an isoform of CDK11 (CDK11/p58), which exists in large amounts only during M phase, inhibits cap-dependent translation through the phosphorylation of Thr255 and/or Ser258 in eIF3F, a subunit of eIF3. The co-immunoprecipitation of CDK11/p58 and eIF3F (Fig. 3) and the synergistic inhibition of cap-dependent translation by co-expression of these proteins (Fig. 6) indicate that CDK11/p58 directly phosphorylates eIF3F.

Even though several functions of CDK11/p58 during M phase have been suggested, none of them revealed that CDK11/p58 functions in translational regulation. Thus, we uncovered a novel function of CDK11/p58 in modulating general translation during M phase. A previous study showed that CDK11/p46 produced in apoptotic cells represses translation by phosphorylating eIF3F [32]. However, the CDK11/p46-mediated phosphorylation sites in eIF3F (Ser46 and Thr119) are different from the CDK11/p58-mediated phosphorylation sites (Thr255 and/or Ser258) (Fig. 6). The difference in residues targeted by CDK11 isoforms (CDK11/p46 and CDK11/p58) is likely attributed to the extended N-terminal region of CDK11/p58 (50 amino acids) compared with CDK11/p46 and/or to the cyclin D3 associated with CDK11/p58, which facilitates the kinase activity of CDK11/p58 [30, 61].

In this study, we demonstrated that the mitotic phosphorylation of eIF3F by CDK11/p58 leads to translational repression (Figs. 7, 8). We also showed that the phosphorylation of eIF3F does not block its interaction with at least some of the eIF3 components (Fig. S5). Moreover, we found that the phosphorylated eIF3F has a sedimentation velocity between eIF3 complex and free eIF3F (Fig. S6). Considering these phenomena, we speculate three plausible mechanisms of translational repression by phosphorylated eIF3F. (1) Phosphorylated eIF3F may compose an immature-nonfunctional eIF3 complex lacking some of eIF3 subunits, which blocks completion of eIF3 complex formation. According to a recently solved structure of the mammalian eIF3 complex, eIF3F is located in the center of the seven-helix bundle composed of the C-terminal α-helices of eIF3C, E, F, H, K and L [59]. In addition, it was suggested that the eIF3A/F/M subcomplex serves as an initial module for helix bundle formation through a study of the *Neurospora crassa* eIF3 complex [62]. Therefore, it is likely that eIF3F plays an important role in the initial step of eIF3 complex formation. Phosphorylated eIF3F seems to facilitate the formation of immature-nonfunctional eIF3 complex since the phosphorylated eIF3F does not exist in free form at both interphase and M phase as shown in the fraction D of Fig. S6. (2) Phosphorylated eIF3F may make a complex with an unknown protein, which in turn induces translational repression during M phase. It should be noted that translational inhibition during M phase reaches up to 50% (Fig. 8b) but the M phase-specific phosphorylation of eIF3 reaches only up to 15% (Fig. 4), and that a homologous gene of eIF3F is not present in yeast (*S. cerevisiae*), which suggests that eIF3F is not essential for translational activation by eIF3. Moreover, overproduction of eIF3F inhibits cap-dependent translation [32] (also see Fig. 5), and knockdown of eIF3F increases cell proliferation [46]. It is noteworthy that an analogous translational repression has been documented in the interferon (IFN)-γ-activated inhibitor translation (GAIT) system even though the GAIT system functions for specific mRNAs. A ribosomal protein L13a is phosphorylated by DAPK–ZIPK kinase axis and released from the 60S ribosomal subunit about 16 h after IFN-γ stimulation, and then the phosphorylated L13a associates with pre-GAIT complex to form the functional GAIT complex which represses translation of specific mRNAs, such as human ceruloplasmin mRNA, containing a GAIT element in the 3′ untranslated region [63, 64]. (3) The immature eIF3 complex described in speculation (1) may associate with a limiting translation factor and inactivates (or sequestrates) the putative translation factor. The detailed mechanism of translational inhibition by the phosphorylated eIF3F remains unknown. A further investigation is in progress to understand the molecular mechanism of translational inhibition by CDK11/p58-dependent eIF3F phosphorylation.

Blocking eIF3F phosphorylation by knockdown of CDK11/p58 (Fig. 2c) or by overproduction of unphosphorylatable eIF3F (Fig. 7) in M phase cells, synchronized by nocodazole treatment, restored translation in M phase by approximately 35% and 50%, respectively, compared with translation in interphase (Figs. 2, 7). The partial restoration, even after blocking the effect of CDK11/p58, is likely attributed to two reasons: (1) other mechanism(s), such as mitosis-specific binding of 14-3-3σ to eIF4F 4B [65] and/or mitosis-specific activation of PKR, which results in phosphorylation of eIF2α [23], may also contribute to the translational repression in M phase; (2) the contribution of translational repression by the toxic effect of nocodazole, which is unrelated to the M phase-specific translation inhibition [60]. The authors suggested that phosphorylation of eIF2α induced by nocodazole treatment inhibits translation regardless of cell cycle [60].

Recently, Tanenbaum and his colleagues showed that M phase-specific translational inhibition occurs using a different cell cycle blocker, RO-3306 (CDK1 inhibitor), and genome-wide ribosome profiling analysis [15]. The authors showed that overall translation efficiency decreases approximately 35% in M phase compared with G1 and G2 phases through ^35^S-methionine labeling of newly synthesized proteins using a cell cycle arrest-and-resumption approach with RO-3306.

In this study, we also performed a cell cycle arrest-and-resumption experiment using double thymidine block and puromycine labeling (Fig. 8). We used this approach to minimize the synchronizing chemical effect in M phase and the stress generation in protein labeling process; that is, the recovery time after the cell cycle arrest is longer in the thymidine block than RO-3306 treatment (9 h vs. 45 min), and the methionine starvation step is not required for the SUnSET method. Through this approach, we found that overall translation decreases to about 50% in M phase cells (Fig. 8b). To our surprise, the translational repression in M phase was not observed at all in the cells ectopically expressing eIF3F(A3A4). The results indicate that strong repression of overall translation occurs in M phase cells under normal conditions and that the phosphorylation of eIF3F by CDK11/p58 plays a major role in the translational repression in M phase. Moreover, the results suggest that the partial restoration of translation in M phase cells, expressing eIF3F(A3A4) and synchronized by nocodazole (Fig. 7b), is attributed to a toxic effect of nocodazole.

Even though mitotic translational repression is a well-known feature of mammalian cells, the physiological roles of translational modulation during cell cycle progression are not clearly understood. We speculate possible roles of translational repression during M phase. (1) Translation repression of some genes during mitosis is needed for cell cycle progression from M to next G1 phase. For example, the specific translational inhibition of Emi1 gene, an inhibitor of anaphase-promoting complex (APC) formation, is required for the activation of APC during M phase, which in turn is needed for cell cycle progression [15]. (2) Translational repression during M phase would minimize the synthesis of aberrant polypeptides encoded in immature pre-mRNAs that are potentially exposed to translational machineries in the cytosol during M phase by the disappearance of the nuclear envelope [66]. (3) The reduction of cap-dependent translation during M phase seems to contribute to IRES-dependent translation by relieving the competition of limited translation factors [67].

An interesting relationship between cap-dependent and IRES-dependent translation during M phase was revealed via investigations into the role of a tumor suppressor gene, 14-3-3σ, in M phase-specific translational regulation [65]. The authors showed that 14-3-3σ is required for M phase-specific translation repression that is in turn required for IRES-dependent translation of CDK11/p58. Knockdown of 14-3-3σ resulted in increased cap-dependent translation and decreased IRES-dependent translation of CDK11/p58 during M phase, which in turn lead to impaired cytokinesis and the accumulation of binucleate cells [65]. Addition of rapamycin, which inhibits cap-dependent translation but not cap-independent translation, to the 14-3-3σ knockdown cells restored the mitotic translation of CDK11/p58 and nullified the aberrant mitotic phenotype. Moreover, the mitosis-defective cell phenotype of 14-3-3σ knockdown cells was partially rescued by ectopic expression of CDK11/p58 [65]. Considering these data and our results presented here, which shows that CDK11/p58 actively reduces cap-dependent translation by phosphorylating eIF3F, the effect of CDK11/p58 expression in 14-3-3σ knockdown cells is, at least in part, attributed to cap-dependent translational repression by CDK11/p58.

Translational regulation by CDK11 and eIF3F pair seems to have deep roots in evolutionary history. Neither CDK11 nor eIF3F homologue exists in *S. cerevisiae*, but both CDK11 and eIF3F homologues exist from *Schizosaccharomyces pombe* (*S. pombe*) to humans. Furthermore, the region corresponding to CDK11/p46 exists in all CDK11 homologues from *S. pombe* to humans, and one of the CDK11/p46 phosphorylation sites in eIF3F (Thr119) exists in all eIF3F homologues (Fig. S7a). In contrast, neither CDK11/p58 nor its target phosphorylation site in eIF3F exists in *S. pombe* (Fig. S7a). This indicates that M phase-specific translational repression by an isoform of CDK11 may not occur in *S. pombe*. Both cyclin D family members and the region in CDK11 corresponding to CDK11/p58 are conserved from *Caenorhabditis elegans* (*C. elegans*) to humans, and one of the CDK11/p58 target sites in eIF3F (Ser258) is also conserved from *C. elegans* to humans (Fig. S7a). This may indicate that cell cycle-dependent translational regulation by a CDK11 isoform (CDK11/p58) functions from *C. elegans* to humans. Other cell cycle-dependent translational regulation mechanisms that have been suggested to function during M phase are not as well conserved evolutionarily as the CDK11 and eIF3F pair. For example, the CDK1 phosphorylation site in eIF4G1, which was suggested to function in M phase-specific translational repression [22], is conserved only in mammals (Fig. S7b). In addition, PKR, which was also suggested to function during M phase-specific translational repression [23], exists only in vertebrates. The evolutionary aspect of M phase-specific translational repression by eIF3F and CDK11/p58 should be investigated experimentally in the future.

### Electronic supplementary material

Below is the link to the electronic supplementary material.

Supplementary file1 (PDF 10232 kb)

Supplementary file2 (PDF 102 kb)

Supplementary file3 (XLSX 13 kb)

Supplementary file4 (XLSX 12 kb)

Supplementary file5 (XLSX 16 kb)

## Abbreviations

CDK11: Cyclin-dependent kinase 11
CCND3: Cyclin D3
eIF3F: Eukaryotic initiation factor 3F
SUnSET: Surface sensing of translation
