# Supplementary material for: A cyclin-dependent kinase, CDK11/p58, represses cap-dependent translation during mitosis
Source: Cell Mol Life Sci. 2020 Feb 6;77(22):4693–708. doi: 10.1007/s00018-019-03436-3 (PMC7599166; doi:10.1007/s00018-019-03436-3)
Supplement: Supplementary file 1 — Supplementary file1 (PDF 10232 kb) [file 18_2019_3436_MOESM1_ESM.pdf]

Supplementary Figure 1

A

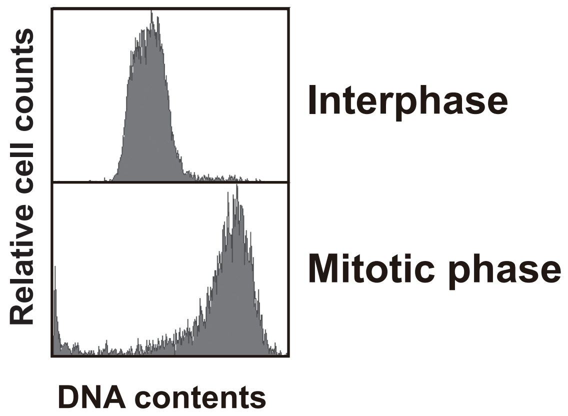

B

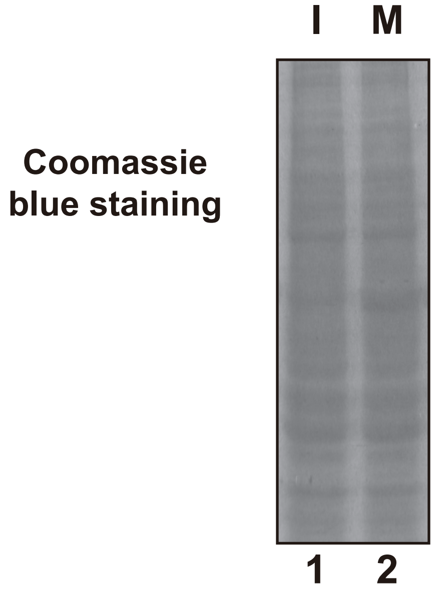

Supplementary Figure 2

A

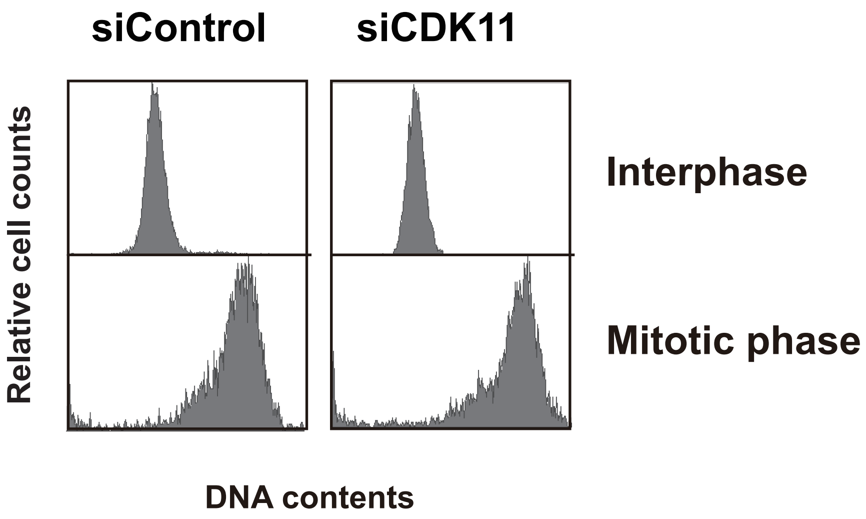

B

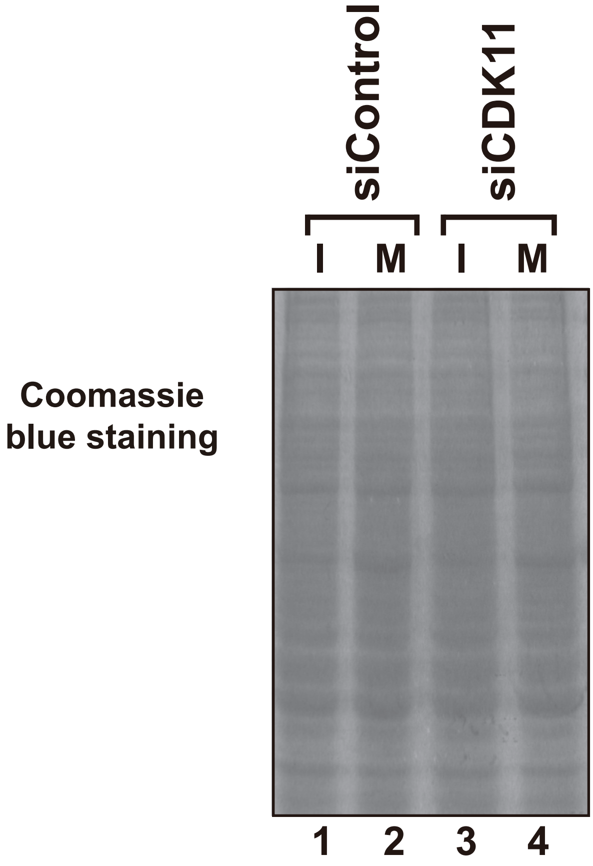

Supplementary Figure 3

A

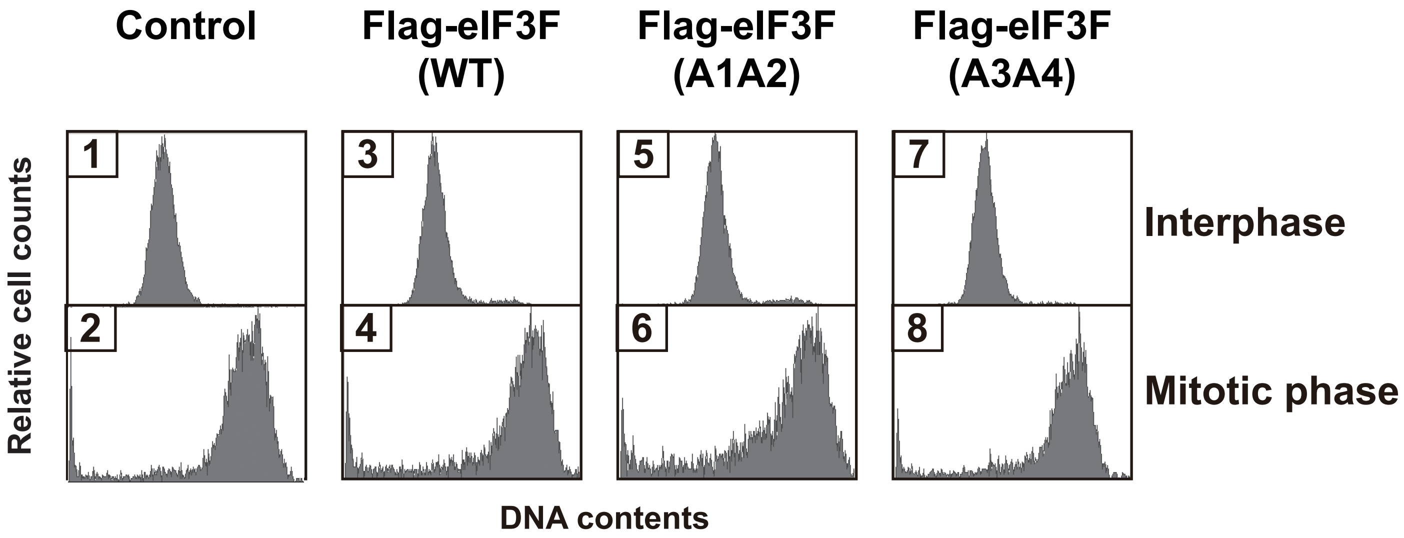

B

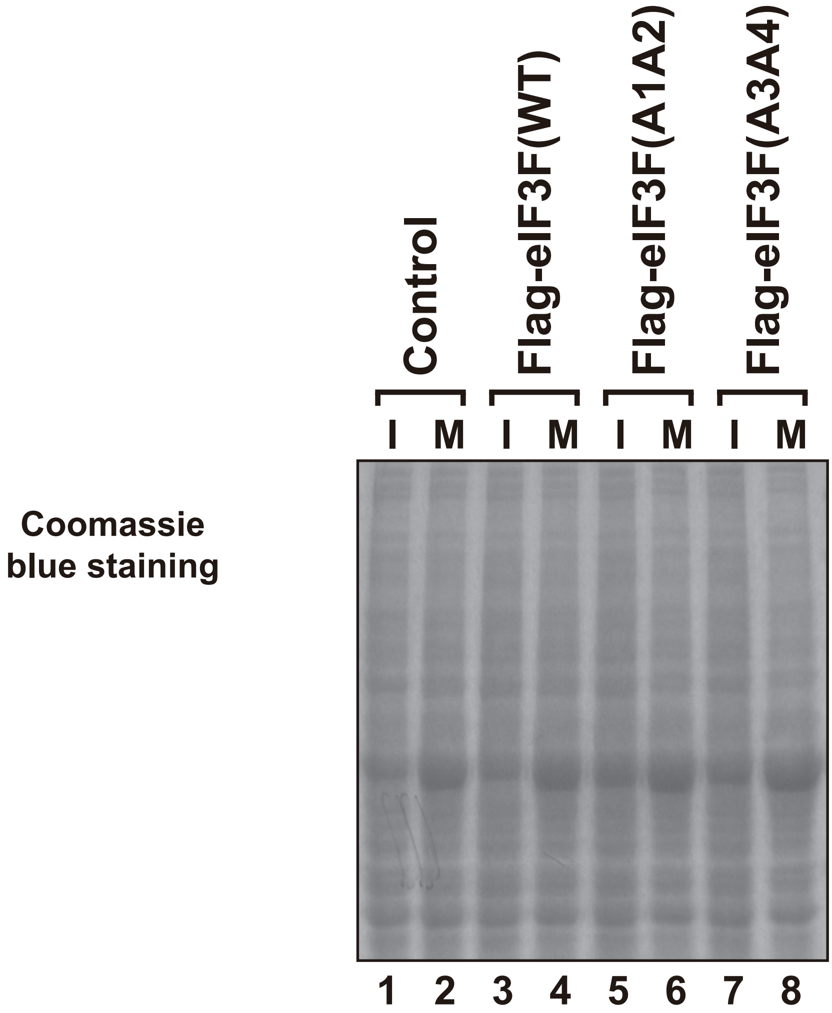

Supplementary Figure 4

A

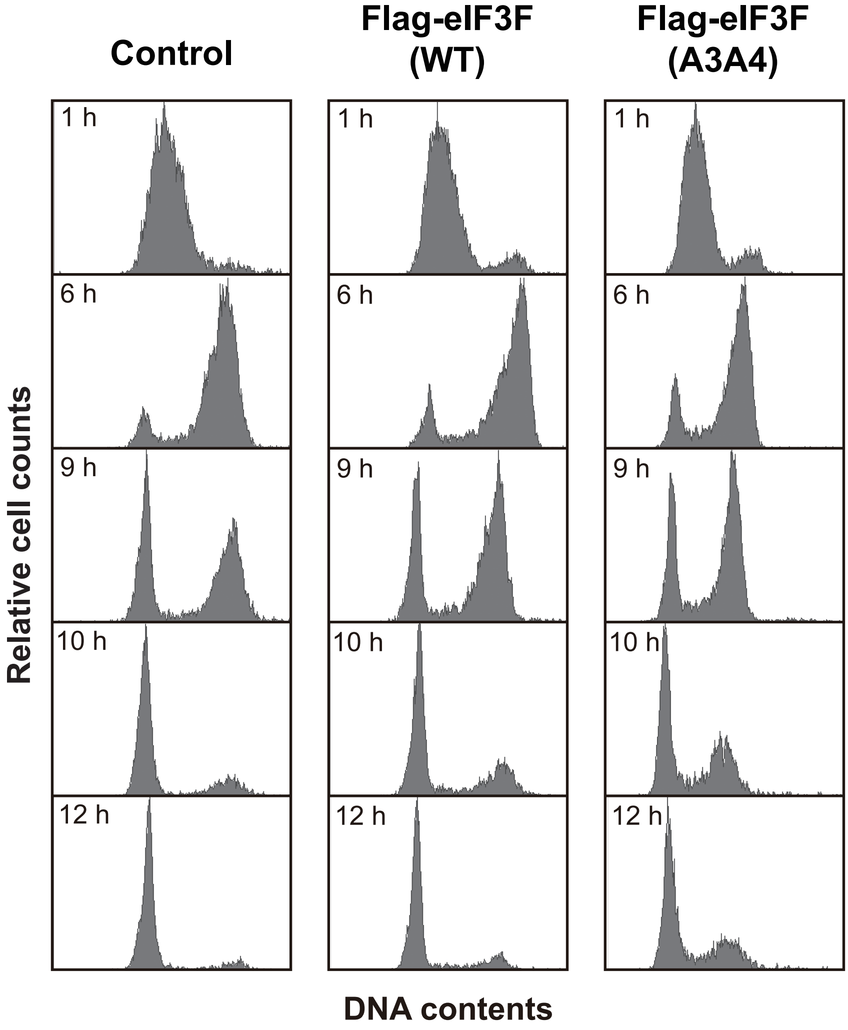

B

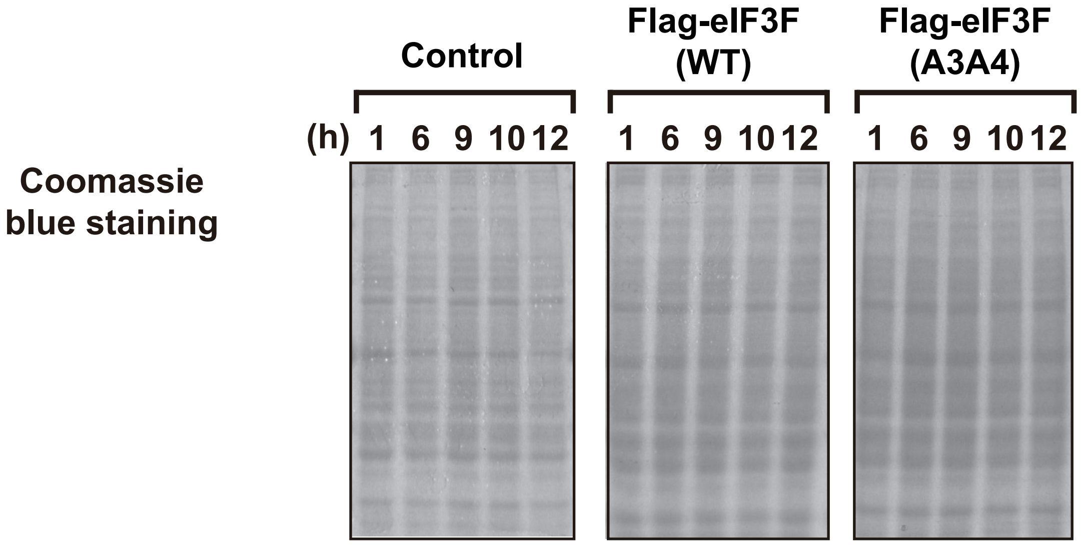

Supplementary Figure 5

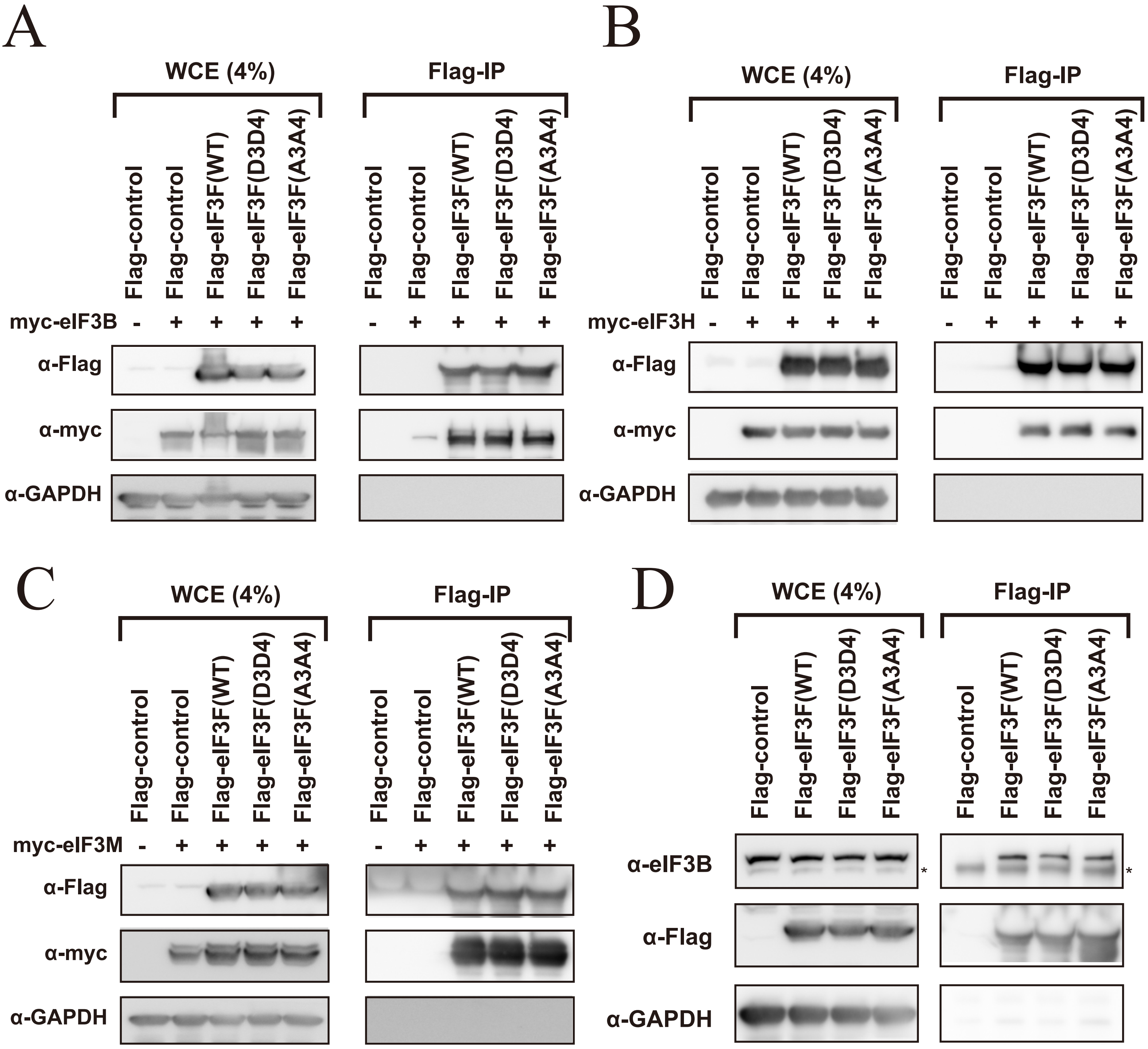

Supplementary Figure 6

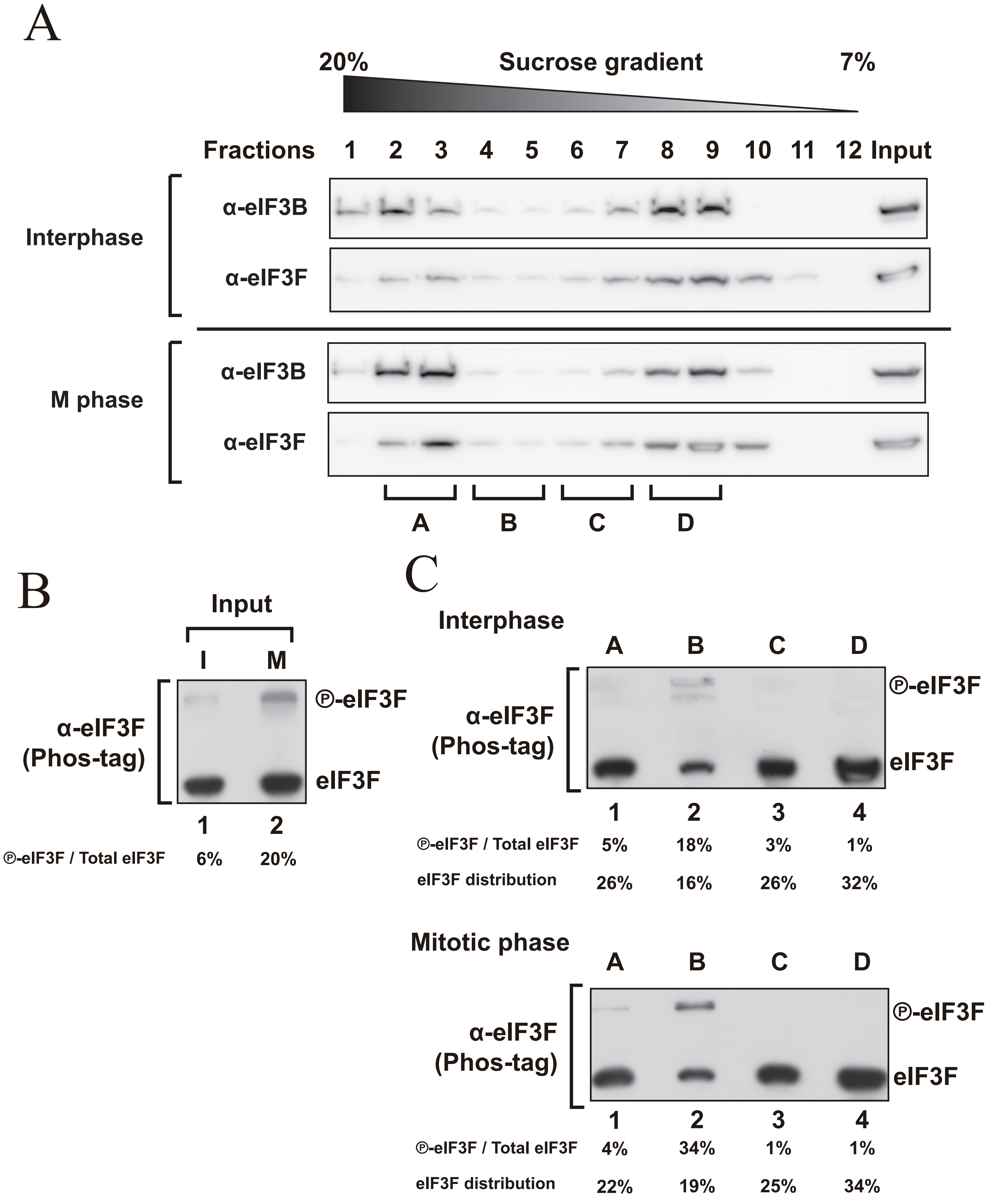

Supplementary Figure 7

A

eIF3F sequence

```
                                Thr119
Human        NEGAARVIGTLLGTVD--KHSVEVTNCFSVPHNESEDEVAVDMEFAKNMYELHKKVSPNE   167
Mouse        NEGAARVIGTLLGTVD--KHSVEVTNCFSVPHNESEDEVAVDMEFAKNMYELHKKVSPNE   171
Zebrafish    NEGASRVIGTLLGTAD--KHSVDVTNCFSVPHNESEDEVAVDMEFAKNMYELHKKVSPSE   83
C. elegans   NTGQEKCMGTLMGYYE--KGSIQVTNCFAIPFNESNDDLEIDDQFNQQMISALKKTSPNE   88
S. pombe     SENNQRVIGTLLGTRSEDGREIEIKSCFAVPHNESSEQVEVEMEYHRAMYHLHLKANPRE   100
             . .  : :***:*  .    .:::..**::*.***.::: :: :: : *     *..* *
```

```
                                                           Ser258
Human        KAYVSTLMGVPGRTMG-V-MFTPLTVKYAYYDTERIGVDLIMKTCFSPNRVIGLSSDLQQ   271
Mouse        KAYVSTLMGVPGRTMG-V-MFTPLTVKYAYYDTERIGVDLIMKTCFSPNRVIGLSSDLQQ   275
Zebrafish    RAYVSSQMGVPGKTVG-V-MFTPLSVKYIYYDTERIGVDLLQRTRASPGRTNGLTTDLAQ   187
C. elegans   RAYLRSKAGIPGAAGPHCAIFNPLRVELAAFPGELVAMQLIEKALDSRRREATLESGLEQ   206
S. pombe     KTYVSSPVGITERLADSC-AFVPTPFTIRDDEAVRSGLKAVAAPKNDPSRLASLFTDLQQ   215
             ::*: :  *:          * *  .          .:. :     .  *   * :.* *
```

B

eIF4G sequence

```
                                          Ser1232
Human        EDRDRGRDAVK---------REAALPPVSPLKAALSEEELEK-KSKAIIEEYLHLNDMKE   1262
Mouse        ED--RGRDPVK---------REATLPPVSPPKAALSVDEVEK-KSKAIIEEYLHLNDMKE   1261
Zebrafish    DNRDRGSRDRDRSKETVKPMAAPALPPPAPVKPAMSEEDLDK-KSKSIIEEYLHINDLKE   1247
C. elegans   SNTDKTDDELTEE---EKAVRAKIMNTVKSDLLEMSSGELSQKELVDSITDYVG------   975
S. pombe     ------------------------------------------------------------   1403
```
