# Supplementary material for: A cyclin-dependent kinase, CDK11/p58, represses cap-dependent translation during mitosis
Source: Cell Mol Life Sci. 2020 Feb 6;77(22):4693–708. doi: 10.1007/s00018-019-03436-3 (PMC7599166; doi:10.1007/s00018-019-03436-3)
Supplement: Supplementary file 2 — Supplementary file2 (PDF 102 kb) [file 18_2019_3436_MOESM2_ESM.pdf]

Supplementary Materials and Methods

Sucrose gradient analysis

HeLa cells were synchronized in either interphase (I) or mitotic phase (M) by double thymidine block or nocodazole treatment. The synchronized cells were washed with cold PBS and harvested with ice-cold lysis buffer [0.1 % NP-40, 40 mM HEPES-KOH (pH 7.5), 100 mM KCl, 1 mM EDTA, 10 mM NaF, 10 mM β-glycerophosphate, 2 mM Na_3VO_4, and 1 mM PMSF]. 7-20 % sucrose gradients (5 ml) were made in lysis buffer without NP-40. Cell lysates were loaded on the top of sucrose gradients. Centrifugation was performed in a Beckman SW55Ti rotor at 45,500 rpm for 13.5 h. After centrifugation, samples were collected in fractions (400 μl each). For Phos-tag analyses, the samples were concentrated by methanol precipitation method.

Supplementary Figure Legends

Figure S1. Synchronization of cells in interphase or M phase. a HeLa cells were synchronized in either interphase (I) or M phase (M) by double thymidine block or nocodazole treatment, respectively. The synchronized cells were harvested and analyzed by flow cytometry. DNA contents were analyzed to determine the cell cycle stage of individual cells. **b** Total proteins in the cell extracts shown in Fig. 2a were resolved by SDS-PAGE and visualized by Coomassie blue staining.

Figure S2. The effect of CDK11 knockdown on cell cycle dependent translation. HeLa cells were transfected with either a control siRNA or a siRNA against CDK11 at 3 h before synchronization. The cells were synchronized in either interphase (I) or M phase (M) by double thymidine block or nocodazole treatment, respectively. **a** The synchronized cells were harvested and analyzed by flow cytometry. DNA contents were analyzed to determine the cell cycle stage of individual cells. **b** Total proteins in the cell extracts shown in Fig. 2c were resolved by SDS-PAGE and visualized by Coomassie blue staining.

Figure S3. The effects of ectopic expression of eIF3F variants on cell cycle dependent translation. HeLa cell lines ectopically expressing eIF3F(WT), eIF3F(A1A2), or eIF3F(A3A4) were established. The cells were synchronized in either interphase (I) or M phase (M) by double thymidine block or nocodazole treatment, respectively. **a** The synchronized cells were harvested and analyzed by flow cytometry. **b** Total proteins in the cell extracts shown in Fig. 7a were resolved by SDS-PAGE and visualized by Coomassie blue staining.

Figure S4. The effect of ectopic expression of unphosphorylatable eIF3F(A3A4) on cell cycle dependent translation. a HeLa cells ectopically expressing eIF3F variants were

synchronized at G1/S boundary by double thymidine block and resumed cell cycle progression by removing the compound. Cells were harvested at the indicated time points after cell cycle resumption and analyzed by flow cytometry. **b** Total proteins in the cell extracts shown in Fig. 8a were resolved by SDS-PAGE and visualized by Coomassie blue staining.

Figure S5. Phosphorylation on eIF3F do not affects eIF3 complex joining. a, b, c Flag-eIF3F variants (WT, D3D4, A3A4) with myc-eIF3B (a), myc-eIF3H (b) or myc-eIF3M (c) were co-transfected to HeLa cells. The Flag-eIF3F variants were immunoprecipitated using an anti-Flag antibody-conjugated protein A agarose resin. Protein levels of Flag-eIF3F variants and myc-eIF3B, myc-eIF3H or myc-eIF3M were monitored by western blotting using anti-Flag and anti-myc antibodies, respectively. GAPDH levels were monitored by western blotting using an anti-GAPDH antibody as an endogenous protein control. **d** Flag-eIF3F variants were transfected to HeLa cells. The Flag-eIF3F variants were immunoprecipitated using an anti-Flag antibody-conjugated protein A agarose resin. Protein levels of Flag-eIF3F variants and endogenous eIF3B were monitored by western blotting using anti-Flag and anti-eIF3B antibodies, respectively. GAPDH levels were monitored by western blotting using an anti-GAPDH antibody as an endogenous protein control. Asterisk (*) means non-specific band.

Figure S6. Sucrose gradient analyses of eIF3F. a Sucrose gradient (7-20 %) analyses were performed with HeLa cells arrested at interphase or mitotic phase. Protein levels of eIF3B and eIF3F in each fraction were monitored by Western blotting using anti-eIF3B and anti-eIF3F antibodies. **b** The levels of unphosphorylated and phosphorylated eIF3F in interphase and mitotic phase cells before sucrose gradient centrifugation were monitored by using Phos-tag SDS-PAGE and Western blotting. The ratios of phosphorylated eIF3F (ⓟ-eIF3F) to total eIF3F

(unphosphorylated and phosphorylated eIF3F) are depicted. The data were similar to the data shown in Fig. 4A. **c** The levels of unphosphorylated and phosphorylated eIF3F in fractions A to D in panel (**a**) were monitored by using Phos-tag SDS-PAGE and Western blotting. The ratio of ⓟ-eIF3F to total eIF3F and the relative level of eIF3F in each fraction are depicted.

Figure S7. Phylogenetic conservation of phosphorylation sites in eIF3F and eIF4G. a Amino acid sequences of eIF3F homologues around Thr119 region (upper panel) and Ser258 region (lower panel) were aligned using the Clustal Omega multiple sequence alignment program. The Thr119 and Ser258 positions are enclosed in boxes. Sequences are from following species: Human (NP_003745.1), Mouse (NP_079620.2), Zebrafish (NP_001186938.1), *C. elegans* (NP_495988.1), *S. pombe* (NP_596298.1). **b** Amino acid sequences of eIF4G homologues in the region containing Ser1232 were aligned using the Clustal Omega multiple sequence alignment program. The Ser1232 position is enclosed in a box. Sequences are from following species: Human (NP_003745.1), Mouse (NP_079620.2), Zebrafish (NP_001186938.1), *C. elegans* (NP_495988.1), *S. pombe* (NP_596298.1).
